# Transcriptomic Module Discovery of Diarrhea-Predominant Irritable Bowel Syndrome: A Causal Network Inference Approach

**DOI:** 10.3390/ijms25179322

**Published:** 2024-08-28

**Authors:** Davide Guido, Fatima Maqoud, Michelangelo Aloisio, Domenica Mallardi, Blendi Ura, Nicolò Gualandi, Massimiliano Cocca, Francesco Russo

**Affiliations:** 1 Data Science Unit, National Institute of Gastroenterology-IRCCS “Saverio de Bellis”, 70013 Castellana Grotte, Bari, Italy; davide.guido@irccsdebellis.it; 2 Functional Gastrointestinal Disorders Research Group, National Institute of Gastroenterology-IRCCS “Saverio de Bellis”, 70013 Castellana Grotte, Bari, Italy; fatima.maqoud@irccsdebellis.it (F.M.); michelangelo.aloisio@irccsdebellis.it (M.A.); domenica.mallardi@irccsdebellis.it (D.M.); 3 Institute for Maternal and Child Health-IRCCS “Burlo Garofolo”, 34137 Trieste, Italy; blendi.ura@burlo.trieste.it; 4 Department of Medicine, Laboratory of Biochemistry, University of Udine, P.le Kolbe 4, 33100 Udine, Italy; nicolo.gualandi@gmail.com; 5 INSERM U1052, CNRS UMR_5286, Cancer Research Center of Lyon (CRCL), 69008 Lyon, France; massimiliano.cocca@inserm.fr; 6 Institute of Hepatology Lyon (IHL), 69002 Lyon, France

**Keywords:** IBS-D, transcriptomics, causal network inference, biomarkers, therapeutic targets

## Abstract

Irritable bowel syndrome with diarrhea (IBS-D) is the most prevalent subtype of IBS, characterized by chronic gastrointestinal symptoms in the absence of identifiable pathological findings. This study aims to investigate the molecular mechanisms underlying IBS-D using transcriptomic data. By employing causal network inference methods, we identify key transcriptomic modules associated with IBS-D. Utilizing data from public databases and applying advanced computational techniques, we uncover potential biomarkers and therapeutic targets. Our analysis reveals significant molecular alterations that affect cellular functions, offering new insights into the complex pathophysiology of IBS-D. These findings enhance our understanding of the disease and may foster the development of more effective treatments.

## 1. Introduction

Functional gastrointestinal disorders (FGIDs) encompass a spectrum of conditions characterized by chronic gastrointestinal (GI) symptoms without identifiable pathological findings on routine diagnostic assessments [1]. Among FGIDs, irritable bowel syndrome (IBS) is the most prevalent subtype [1]. IBS is a multifactorial disorder [2] marked by abdominal pain, altered stool consistency and frequency, and the absence of detectable biochemical or structural anomalies [3]. IBS is classified in four subtypes based on stool patterns: IBS with constipation (IBS-C), IBS with diarrhea (IBS-D), IBS with mixed bowel habit (IBS-M), and IBS unclassified (IBS-U) [4].

This study is primarily focused on IBS-D, which, according to multiple sources, is the most common subtype, followed by IBS-C and IBS-M. One study in an adult population found that IBS-D accounted for approximately 40% of cases, IBS-C for 35%, IBS-M for 23%, and IBS-U for 8% [5]. Interestingly, this functional disorder also affects pediatric populations [6]. Due to the absence of objective diagnostic biomarkers [7], these percentages may vary depending on the study population, the diagnostic criteria used (e.g., Rome III vs. Rome IV), and the geographical region [8]. Nevertheless, IBS-D consistently emerges as the most common subtype across various studies and regions.

Despite its prevalence and clinical impact, the pathophysiology of IBS-D remains poorly understood [9]. Complex diseases like FGIDs are thought to arise from a combination of molecular changes that disrupt cellular function [10]. Advances in omics technologies (e.g., genomics, transcriptomics, and proteomics) [11] have significantly contributed to our understanding of the fundamental mechanisms underlying these conditions [12].

Considerable research has been conducted on IBS and the role of the gut microbiota but our understanding of the biochemical, metabolic, functional, and inflammatory mechanisms underlying IBS-D remains limited [13]. Human studies are often restricted by the limited availability of biopsies for microstructural and molecular analysis [13]. Additionally, there is a lack of robust animal models that fully replicate the pathophysiology of IBS, particularly IBS-D [14]. Thanks to the availability nowadays of well-established experimental protocols and the reduced costs of omics platforms, large quantities of omics data are being generated [14] and made accessible through public databases (e.g., Gene Expression Omnibus) [15]. RNA sequencing (RNA-seq) technology, for example, is a crucial tool for defining the transcriptome landscape from isolated cells, tissue sections, or biopsy samples [16].

In this context, pathway enrichment analysis is essential for identifying active biological processes [17,18,19,20], elucidating disease-associated molecular pathways, devising novel therapeutic strategies, and delineating diagnostic biomarkers [21,22]. Integrating genomic expression data with biomolecular interaction databases, such as Reactome [23] or KEGG [24], offers significant advantages, especially in the big data era. This approach is essential to understand the disease complexity in each patient [12,25]. Consequently, network biology, aimed at enhancing our understanding of complex biological systems, has gained considerable interest in recent years [26]. Identifying pathways that drive disease-specific expression signatures can reveal “hidden nodes,” which, although not differentially expressed, play a crucial role in connecting differentially expressed genes within complex networks [27,28].

In this framework, the modular structure of biological networks provides critical insights into the connections and interactions between elements within a network. A disease module represents a collection of cellular components whose disruption can lead to a disease phenotype. Notably, these modules are identified as clusters of highly interconnected genes that show significant differences in expression between diseased and control cells [27]. Understanding these modules may help to elucidate disease mechanisms and biological functions, particularly during disease progression [29,30]. Furthermore, there is growing interest in employing inference analysis to model and quantify cause–effect relationships within modules, particularly from a multivariate statistical perspective in the context of experimental perturbation [31,32].

Given the complex and multifactorial nature of IBS-D, driven by both genetic and environmental factors, a causal network-based approach utilizing graph theory [33,34] and structural equation modeling [31] could be beneficial. This approach enables transcriptomic interactions, such as active disease modules and their associated communities, to be explored and inferences about their effects drawn. This study aimed to investigate the transcriptomic complexity of IBS-D using a graph theory-SEM integrated approach, applying network model theory to identify an IBS-D active module and its associated communities and explore/infer their effects. This innovative approach may provide new insights into the molecular mechanisms underlying IBS-D.

## 2. Results

### 2.1. Preprocessing and Dataset Selection

The gene expression profiles from two RNA-seq datasets, GSE146853 and GSE166869, derived from intestinal biopsies, were obtained from the NCBI Gene Expression Omnibus (GEO) database. A total of 164 raw FastQ files were processed, representing 38 IBS-D patients and 44 healthy controls. The average sequencing coverage per sample was 42.0 million reads (range: 28.3–68.1 million) for the GSE146853 dataset and 25.3 million reads (range: 3.1–44.7 million) for the GSE166869 dataset (Table S1). Additionally, the average ribosomal RNA (rRNA) content was 15% (range: 11.2–21.7%) for GSE146853 and 77.5% (range: 22.7–99.2%) for GSE166869 (Table S1). Due to insufficient quality control, the GSE166869 dataset was excluded from further analysis. The 35 samples collected at time point 1 (T1) from the GSE146853 dataset were subsequently analyzed. The results are summarized in Table 1.

### 2.2. Differentially Expressed Genes (DEG) and Reactome Enrichment Analyses

**Figure 1:**
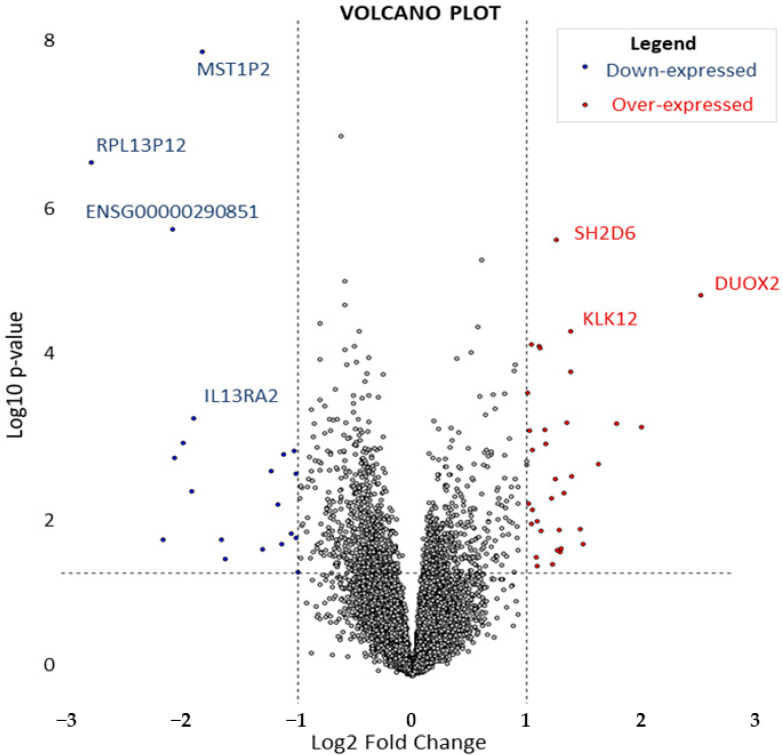
Volcano plot: under-expressed (blue) and over-expressed (red) genes where *p* value < 0.05. Genes marked with gene name or ENSEMBL ID are the most statistically significant.

Preliminary principal component analysis (PCA) indicated that disease status was not associated with transcriptomic variability, as reflected in the first two principal components, regardless of their complexity (Figure S1). To further explore the transcriptomic complexity, a DEG analysis was conducted as an initial step, comparing the 35 samples (17 IBS-D patients and 18 healthy controls). This analysis identified 54 significant DEGs (*p*-value < 0.05), 34 being upregulated (log2FoldChange ≥ 1) and 20 downregulated (log2FoldChange ≤ −1). The identified DEGs are detailed in Table 2 and visualized in the volcano plot (Figure 1).

**Figure 2:**
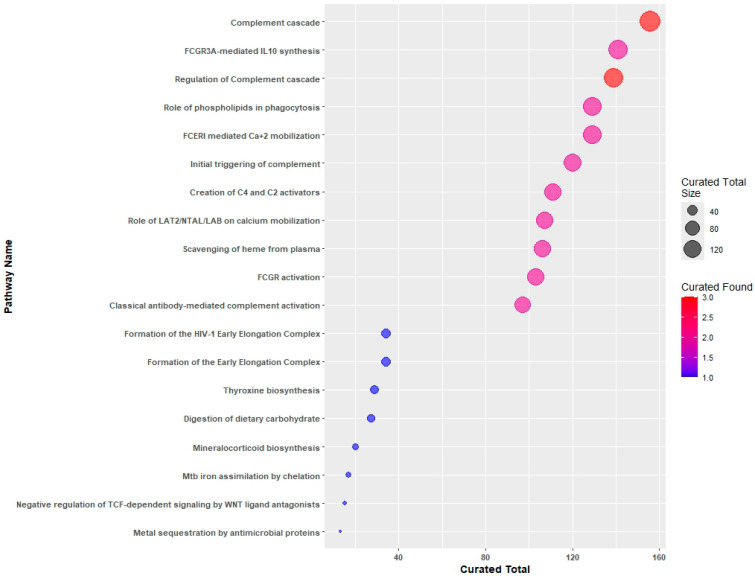
Reactome pathways identified by the 54 differentially expressed genes (DEGs) are depicted as dots. The size of each dot represents the number of genes included in the pathway, while the color corresponds to the number of DEGs contained within the gene set of the pathway.

The DEGs detected by their ENSEMBL IDs were used to identify pathways in Reactome analysis tools that include at least one DEG, as detailed in Table S2 and illustrated in Figure 2.

### 2.3. Statistical Analysis

#### 2.3.1. SEM-Based Gene Set Analysis (SEMgsa) 

In the first step, an SEMgsa was performed on notable genes—without considering the newly discovered transcripts by RNA-seq analysis. Table 3 displays the results of the procedure. Notably, the pathways involved in the complement system—including the generation of C4 and C2 activators, classical antibody-mediated complement activation, the complement cascade, and its regulation—show significant inhibition (*p* < 0.05).

As shown in Table 4, 22 unique DEGs were identified. The pathways complement cascade and regulation of complement cascade included the highest number of DEGs, involving eight and seven genes, respectively. Consequently, these DEGs were also used as seed genes for the Steiner tree (ST) identification procedure. 

#### 2.3.2. Analysis of the Active IBS-D Module

**Figure 3:**
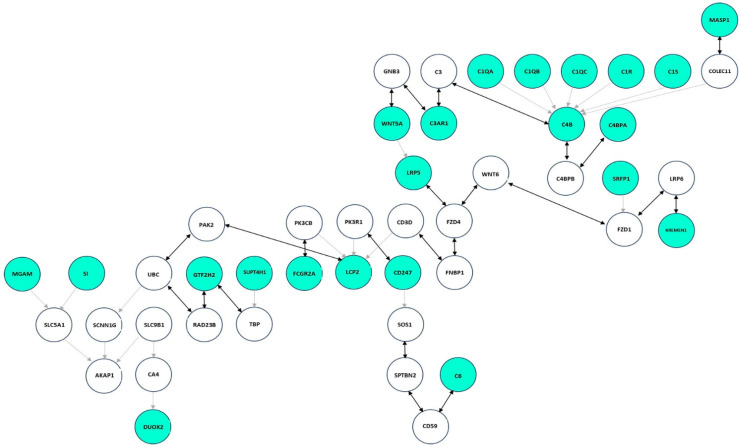
Steiner tree graph of IBS-D from Reactome and GEO data. Seed nodes are colored aquamarine, connectors white, and bidirectional edges are colored black. The graph is drawn from DEGs (seed genes) mapped on the Reactome interactome, i.e., the union of all Reactome pathways, in relation to a weighting procedure based on Fisher’s r-to-z transformation.

Figure 3 shows the ST graph for IBS-D, generated by integrating Reactome pathways [23] and GEO data. The graph consists of 46 nodes and 45 edges. The 22 seed genes corresponding to DEGs are highlighted in aquamarine, and the 24 connector nodes in white. Bidirectional edges are represented in black. 

**Figure 4:**
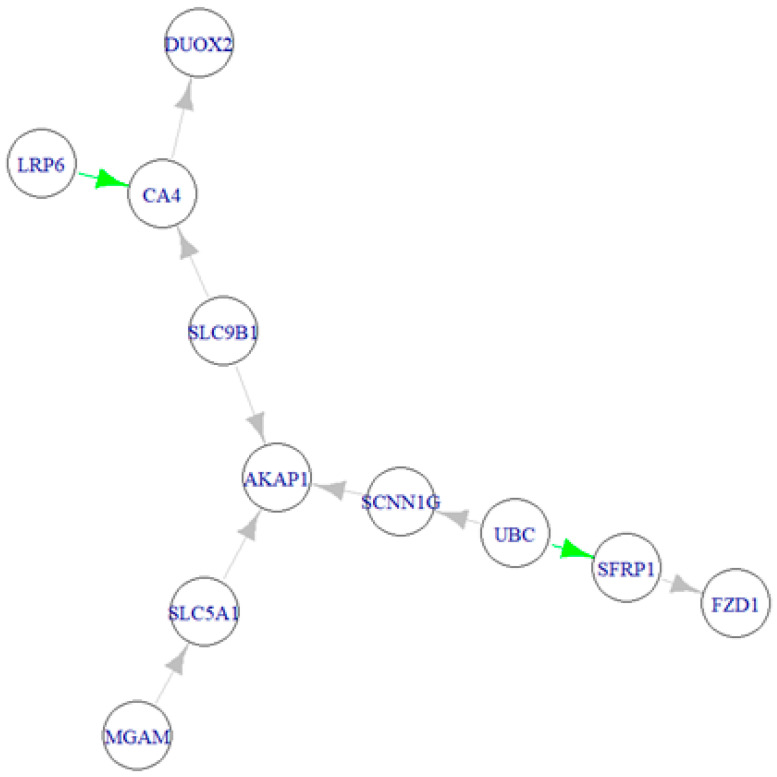
The active IBS-D module (i.e., the optimal IBS-D DAG was derived using the SEMgraph fitting strategy. Added edges are highlighted in green, while gray edges represent those retained from the original IBS-D graph.

Figure 4 illustrates the active IBS-D module, represented as the optimal IBS-D Directed Acyclic Graph (DAG), derived using a fitting strategy based on Structural Equation Modeling (SEM) and graph theory, as described in the methods section implemented in the R/SEMgraph packages [25]. In this approach, we developed a data-driven model search strategy, relying solely on data without validation against a reference network. Notably, Figure 4 highlights the addition of two edges: LRP6 → CA4 and UBC → SFRP1.

Table 5, Table 6 and Table 7 present the results of the SEM fitting analysis. Notably, both the IBS-D and control models yielded similar findings for the significant CA4 → DUOX2 effect (activation). However, there were contrasting results regarding the significant/suggestive SLC9B1 → CA4 effect—activation observed within IBS-D samples, while no activation was seen in the control samples. Similarly, the SCNN1G → AKAP1 effect showed non-activation in IBS-D samples but activation in control samples (see Table 5). 

Regarding perturbation effects, CA4 (upregulated) and SFRP1 (downregulated) showed significant changes, while UBC was upregulated in a suggestive manner (0.05 < *p* < 0.10) (see Table 6).

Regarding edge differences between groups, the SLC9B1 → CA4 and UBC → SCNN1G effects were significantly upregulated, whereas the SLC9B1 → AKAP1, SLC5A1 → AKAP1, and SCNN1G → AKAP1 effects showed significant downregulation (see Table 7).

**Figure 5:**
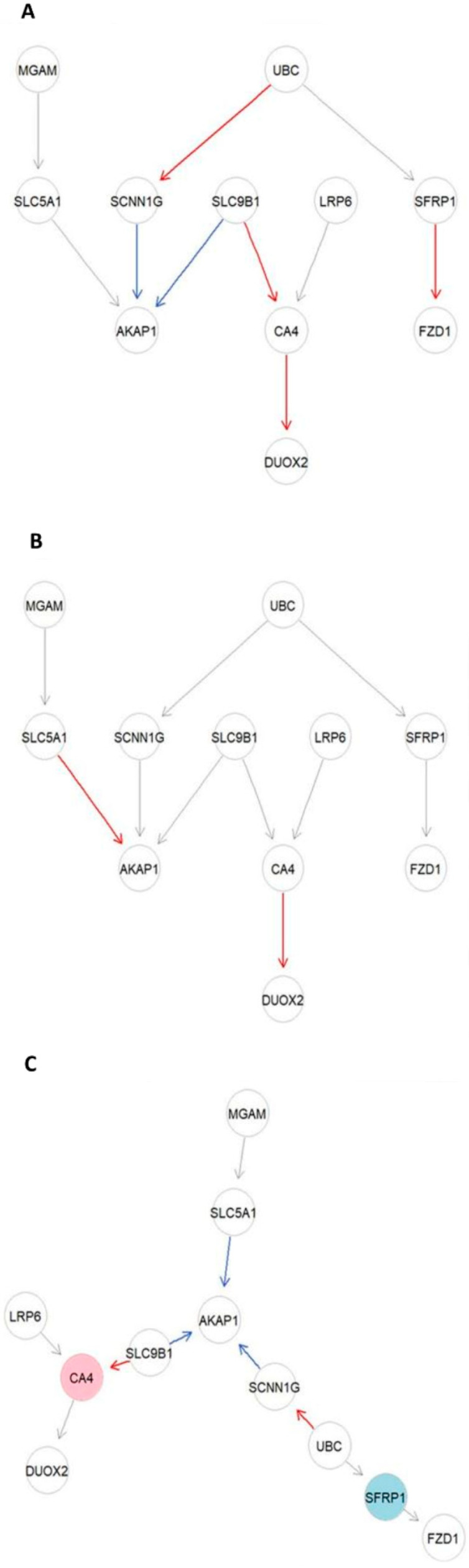
Final active IBS-D modules after SEM fitting. The final active IBS-D modules, derived from structural equation modeling (SEM) fitting, are presented as follows: Nodes and Edges: pink nodes: activated; light blue nodes: inhibited; Edges: red edges: significant direct effects (*p*-value < 0.05) with a positive effect (b effect > 0). Blue edges: significant direct effects (*p*-value < 0.05) with a negative effect (b effect < 0). Gray edges: non-significant effects. (**A**) IBS-D Module. This diagram represents the IBS-D module, including only IBS-D samples. The module was obtained using SEM fitting with constrained Gaussian graphical modeling (CGGM), utilizing a directed acyclic graph (DAG) node-wise Lasso procedure and de-biasing asymptotic inference. In this module, the gene effects are conditional, representing the expected normalized gene expression variation per unit increase of the gene, while keeping other genes fixed. (**B**) Control module. This diagram illustrates the control module, consisting solely of control samples. It was derived using the same SEM fitting method as for the IBS-D module (CGGM with DAG node-wise Lasso and de-biasing asymptotic inference). (**C**) Final active IBS-D module (node and edge perturbation). This module shows the final active IBS-D network with node and edge perturbations. It was obtained using SEM fitting with CGGM, incorporating node-wise Lasso and de-biasing asymptotic inference.

To better interpret the results of the SEM analysis, Figure 5 presents the three final graphs of the active IBS-D module: one for IBS-D samples (Figure 5A), one for control samples (Figure 5B), and a combined “node and edge perturbation” model illustrating the differences between the groups (Figure 5C). In these graphs, edges are color-coded based on their significance: significant direct effects (*p*-value < 0.05) in red (estimate > 0) or blue (estimate < 0), In contrast, non-significant edges are shown in gray. In Figure 5C, nodes are also color-coded, with pink nodes indicating activation and light-blue nodes indicating inhibition. Notably, no changes in causal relationships were observed.

#### 2.3.3. Gene Communities Detection

**Figure 6:**
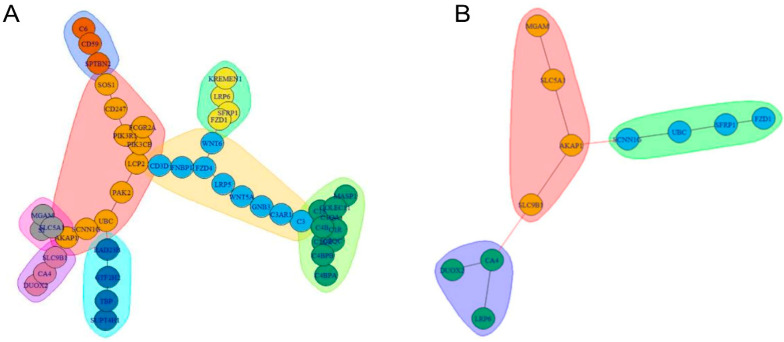
The gene community structure obtained through clustering is illustrated as follows: genes are represented by circles, and community clusters are depicted by colored sets. The topological clustering procedure was performed by applying the walk-trap community detection algorithm (WTC), generating as many clusters as needed to cover the whole input network. (**A**) Clustering performed on the Steiner Tree graph. (**B**) Clustering performed on the optimal DAG obtained from SEMgraph fitting strategies.

Regarding the gene community structure identified through clustering, eight topological clusters (modularity = 0.668) were found in the Steiner tree graph (Figure 6A), and three clusters (modularity = 0.455) were identified in the optimal DAG (Figure 6B). The genes within the eight topological clusters of the Steiner tree are detailed in Table S3.

In the active module, the clusters are organized as follows: the first cluster includes DUOX2, CA4, and LRP6; the second cluster comprises MGAM, SLC5A1, AKAP1, and SLC9B1; and the third cluster consists of SCNN1G, UBC, SFRP1, and FZD1.

#### 2.3.4. Characterization of the Gene Communities

**Figure 7:**
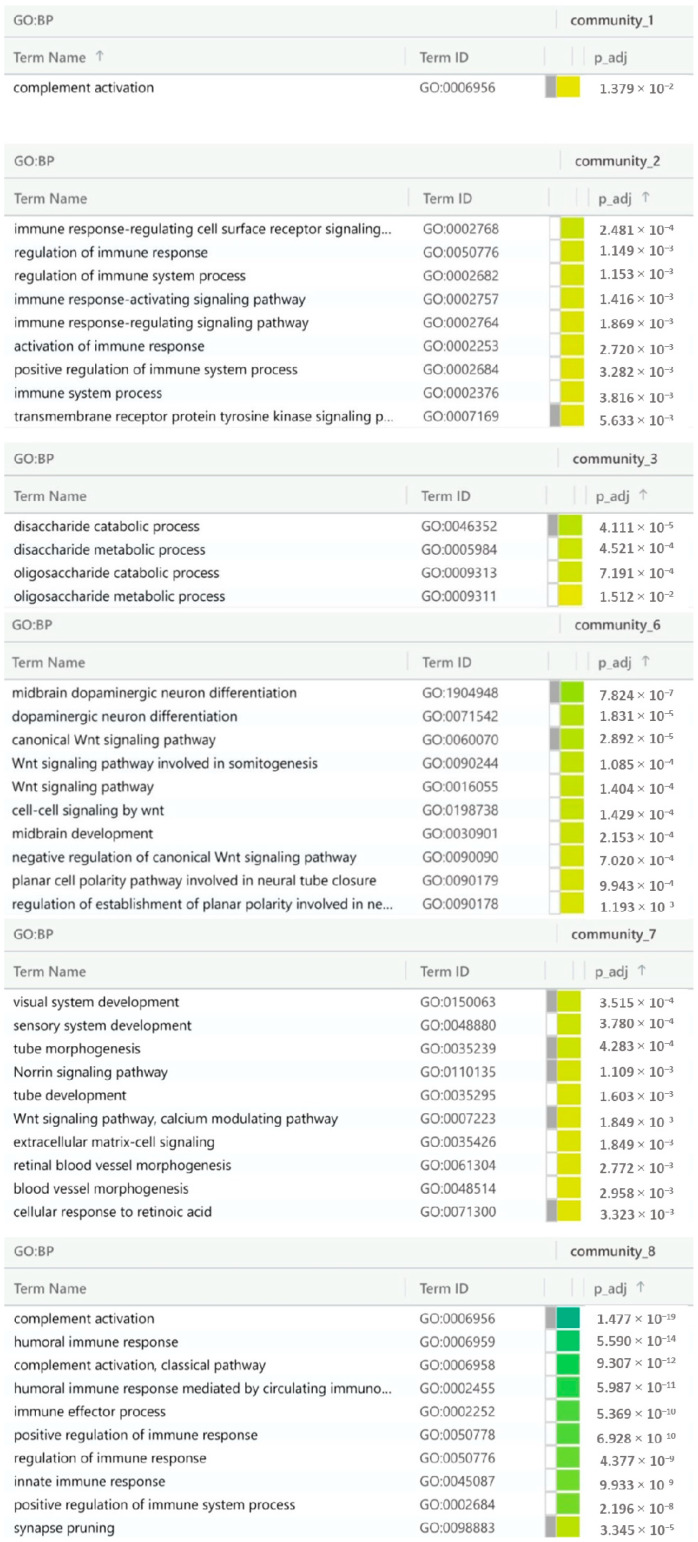
Gene ontology results of ST communities obtained with gprofiler tools. The genes in communities 4 and 5 were not enriched in any GO term pathway. Abbreviations used: BP—biological processes, Term name—GO term name, Term_id—GO term identification, adj *p*-value—adjusted *p*-value.

The results of the characterization of the eight ST-related communities are presented in Table 8 and Figure 7. For detailed information on the genes belonging to these eight topological ST clusters, refer to Table S3.

**Figure 8:**
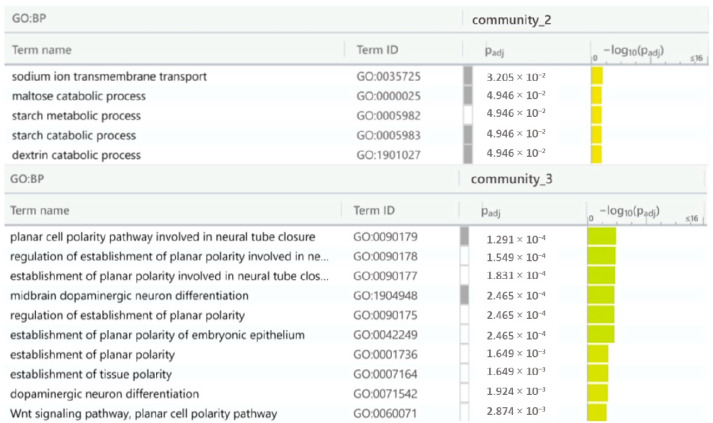
Gene ontology results of IBS-D module communities obtained with gprofiler tools. The genes in the first community were not enriched in any GO term pathway. Abbreviations used are BP—biological processes, Term_name—GO term name, Term_id—GO term identification, and adj *p*-value—adjusted *p*-value.

The active IBS-D module’s communities were characterized by inputting the list of genes appertaining to the three communities; in this study, a ‘community number’ was attributed to each community for its identification. Notably, in the active module, the first community contained the DUOX2, CA4, and LRP6 genes; the second the MGAM, SLC5A1, AKAP1, and SLC9B1 genes; and the third one included the SCNN1G, UBC, SFRP1, and FZD1 genes. The results of the enrichment are shown in Figure 8 and Table 9. 

## 3. Discussion

Research on IBS-D remains limited, and the biochemical, metabolic, functional, and inflammatory mechanisms underlying this disorder are still not fully understood. Current guidelines for managing IBS-D patients do not recommend endoscopic investigation with biopsy sampling, which makes it challenging to conduct detailed tissue and molecular investigations [35,36]. This study uses existing RNA-seq data to bridge these gaps and highlights the importance of integrating complementary data using advanced statistical methods. This approach aims to probe the functional and molecular mechanisms of IBS-D pathophysiology and may potentially help to identify molecular targets for developing and validating therapeutic strategies within a comprehensive, personalized treatment framework.

In biology and medicine, a significant challenge is posed by the need to identify crucial relationships or disrupted disease modules. Greedy algorithms are vital to identify the “active” disease module [35]. This study successfully identified and statistically matched the active module of IBS-D through gene network analysis, which revealed changes in molecular activity [35,37]. 

Using SEM to manage complex systems as multivariate networks, we combined network analysis with causal inference. This approach ensures robustness and reproducibility through a data-driven methodology [25]. Traditional statistical methods such as regression modeling often struggle to simultaneously handle multivariate, inferential, and graph theory aspects, while SEM effectively uncovers subtle gene dysregulations [25].

Our preliminary unsupervised PCA analysis of the 35 samples from GSE146853 indicated that IBS-D status was not associated with transcriptomic variability, suggesting that IBS-D is a functional disorder without significant structural or phenotypic alterations [1]. Topology-based enrichment analysis, a powerful method for identifying enrichment in gene expression data [38], revealed a significant inhibition of seven pathways related to the complement system. Notably, intestinal epithelial cells are the primary source of complement component production in the gastrointestinal tract [39], and the complement system is crucial for systemic microbial defense [40]. This system plays a key role in detecting and eliminating harmful bacteria, maintaining tissue homeostasis, and preventing infections [41]. Several studies have highlighted the interactions between intestinal microbiota dysbiosis and innate immune system activation in IBS [42], leading to inflammation, increased intestinal permeability, and alterations in the neuroendocrine system [43]. Increased levels of β-defensin-2 in IBS patients support the involvement of immune dysfunction in IBS [44,45]. Dysbiosis, an imbalance in gut microbes, is strongly linked to intestinal inflammation and GI disorders [46], particularly in IBS-D [47]. Elevated zonulin levels, indicative of an increased intestinal permeability or “leaky gut”, facilitate bacterial translocation and exacerbate IBS-D symptoms [47].

Although the role of the intestinal complement system in irritable bowel disease (IBD) is well-documented [48], its direct impact on IBS-D remains uncertain. It is hypothesized that, like in IBD, dysregulation of the complement system in IBS-D impairs intestinal barrier function and contributes to dysbiosis. Understanding the interplay among gut microbiota, the complement system, and zonulin activation could provide valuable insights into IBS-D pathophysiology and highlight potential therapeutic targets [49]. Modulating the gut microbiota through probiotics, prebiotics, or dietary interventions, along with targeting the complement system and zonulin pathways, could offer novel strategies for managing IBS-D and improving patient outcomes [50].

Regarding the DEG analysis, classical negative binomial analysis identified DUOX2 and SH2D6 as upregulated DEGs. DUOX2, which encodes dual oxidase 2, is involved in hydrogen peroxide production, which is crucial for mucosal defense and gut microbiota regulation. Elevated DUOX2 expression is associated with inflammatory conditions and microbial imbalances in animal models [51]. SH2D6, or SH2 domain-containing protein 6, plays a role in immune signaling pathways and is a marker for immune CD45+ Tuft-2 cells with antibacterial function [52]. The overexpression of DUOX2 and SH2D6 observed in IBS-D patients aligns with findings related to dysbiosis and complement pathways. The complement system, a component of the innate immune response, is crucial for maintaining gut homeostasis and controlling microbial populations in IBS-D patients, while dysregulation leads to microbial imbalance [41].

In the active IBS-D module, a Steiner tree algorithm was used to discover the transcriptomic network structure through a graph-based data-driven approach. This approach compactly connected “seed” nodes with additional nodes [25], building a more complex and causal network architecture. SEM fitting identified critical gene interactions and regulatory mechanisms, revealing two dysregulated genes: Carbonic Anhydrase 4 (CA4) and Secreted Frizzled-Related Protein 1 (SFRP1). The SEM approach detected these dysregulations, which were not identified using classical negative binomial analysis.

CA4, a zinc metalloenzyme, is involved in numerous physiological functions, including respiration, pH balance, and metabolic processes [53]. In IBD animal models, CA4 inhibition mitigates visceral pain and protects against colon damage [54]. This suggests that CA4 inhibitors could be potential treatments for visceral pain and colon protection in IBS-D. 

SFRP1, a Wnt pathway antagonist, is regulated by promoter hypermethylation in colon and gastric tumors [55,56], and similar epigenetic regulation may occur in IBS-D, affecting inflammatory processes [57]. 

Among the IBS-D active module genes, SLC9B1, SLC5A1, and SCNN1G are involved in proton, electrolyte, and glucose transport. Disruptions in these processes can increase macromolecule permeability [58,59], leading to mucosal inflammation and epithelial barrier disruption, which may trigger innate immune responses and complement activation [50]. This suggests a water and electrolyte absorption disruption in IBS-D, resulting in increased epithelial permeability.

The A-kinase anchoring protein 1 (AKAP1) was involved in three downregulated effects (SLC9B1→ AKAP1, SLC5A1 → AKAP1, SCNN1G → AKAP1). AKAP1 recruits protein kinase A (PKA) and other signaling proteins to the external mitochondrial membrane, playing a crucial role in mitochondrial function, metabolic homeostasis, and disease onset and progression [60,61]. 

The interaction between SLC9B1 (NHE1) and CA4 suggests potential pH regulation alterations, while the interaction between UBC and SCNN1G involves ubiquitin C and ENaC, affecting sodium ion transport and fluid reabsorption [62,63]. 

In summary, the reported disease module analysis highlights the complexity of IBS-D pathology and suggests novel therapeutic strategies involving gut microbiota modulation, Wnt pathways, complement system targeting, and the regulation of water and electrolyte absorption. The methodology also identified biological sub-networks through pathway visualization, revealing that three of the eight Steiner tree-related communities are involved in the immune system. Notably, the complement pathway and the third community—enriched in di- and oligosaccharide metabolic processes—were relevant to IBS symptom management [64]. The sixth and seventh communities showed enriched Wnt signaling pathways, with direct correlations to the gut-brain axis [65,66]. The fourth and fifth communities were too small to offer any detailed explanation. Thus, concerning the IBS-D active module, the second and third communities were involved in metabolic processes and gut-brain axis correlations, by confirming the result from the Steiner tree communities analysis. Overall, the two community analyses confirm the IBS-D complexity and the involvement of both immune system and gut–brain axis.

## 4. Materials and Methods

### 4.1. Dataset Description and Study Workflow

**Figure 9:**
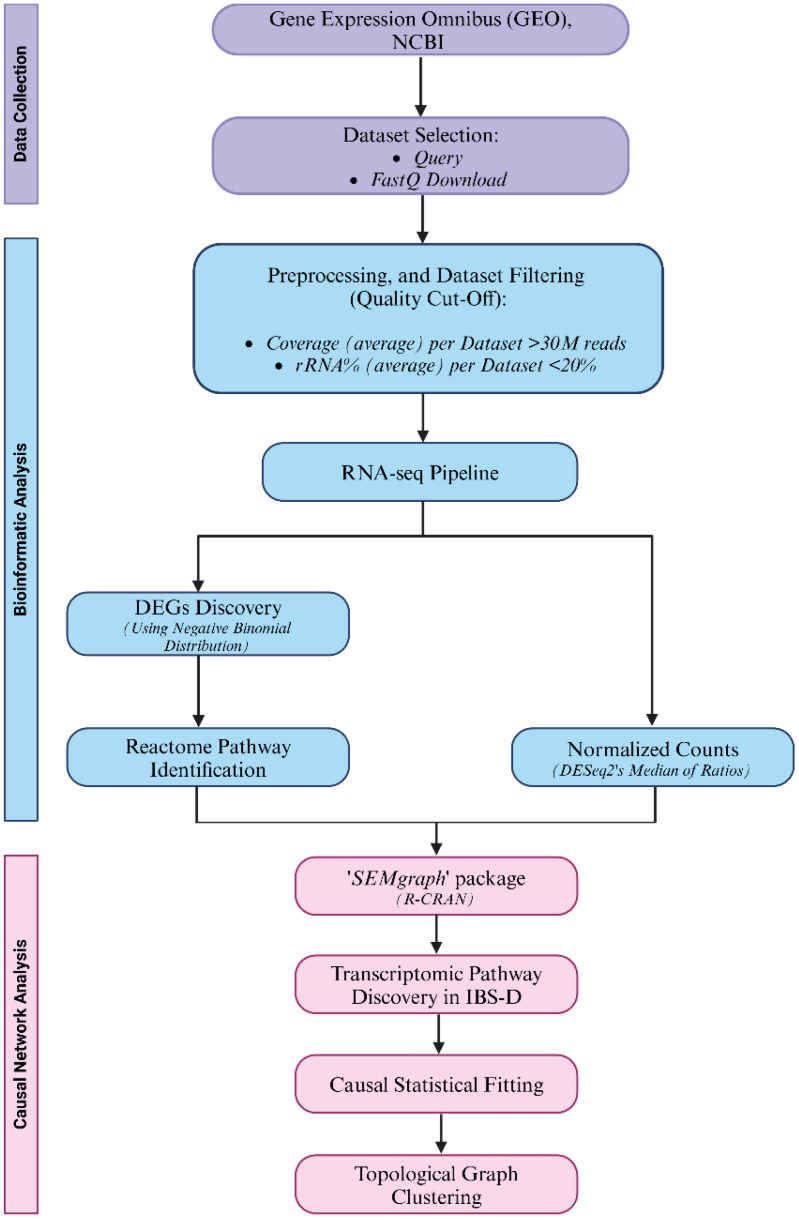
The study workflow of the proposed approach. Data collection steps are highlighted in purple, RNA-seq bioinformatic analysis in light blue and the causal network analysis step is highlighted in pink.

To select RNA-Seq gene expression datasets from the public NCBI GEO, the query ‘(irritable bowel syndrome) AND (diarrhea OR IBS-D) AND (transcriptome OR RNA-seq) AND biopsy AND Homo Sapiens’ was used (assessed on 13 May 2024). This query yielded two bulk RNA-seq poly-A datasets, identified as GSE146853 and GSE166869. After verifying that these datasets contained experimental case–control data and that the data were generated using the Illumina sequencing platform (Illumina Inc., San Diego, CA, USA), the raw paired-end Fastq files were downloaded. To obtain the IBS-D active module and cluster communities, we followed the workflow depicted in Figure 9.

### 4.2. RNA-seq Pipeline and Dataset Quality Control Cut-Off

Fastq file coverage, expressed in millions of reads per sample, was assessed using FastQC v0.11.8 [67]. For RNA-seq analysis of complex eukaryotic transcriptomes, achieving 20–30 million reads per sample is crucial for comprehensive coverage, accurate gene expression quantification, and the detection of low-abundance transcripts [68,69]. A quality control cutoff of greater than 30 million reads per dataset was established to exclude datasets with insufficient coverage.

The ribosomal RNA (rRNA) percentage was computed using the Sequence Expression Analyzer (Seal) from BBtools v39.01 [70]. Typically, rRNA constitutes 60–90% of total RNA. A high rRNA content can negatively impact RNA-seq analysis by dominating the sequencing output, reducing the effective depth of mRNA sequencing, and impeding the detection of rare transcripts. Therefore, a quality control cutoff of less than 20% of the average rRNA percentage per dataset was established to exclude datasets with high rRNA content.

Subsequently, to remove sequencing adapters and low-quality bases, Illumina 50 bp paired-end reads from the Fastq files were trimmed using Trim Galore v0.6.10 [71]. The reads were then mapped to the GRCh38 primary assembly, downloaded from GENECODE version 35 [72], using the STAR aligner (version 2.7.0f) [72,73]. The raw gene counts matrix was obtained using Subread feature Counts (v2.0.2) [74], utilizing the GTF file from the primary assembly. The complete RNA-seq pipeline is available at https://github.com/MichelangeloAloisio/mRNAseq_pipeline (accessed on 2 January 2024) and the full matrix containing gene counts for all the 35 samples, is available on request.

### 4.3. DEGs Analysis and Reactome Enrichment Analysis 

To preliminarily assess the raw variability of the data, we performed a principal component analysis (PCA) on log-transformed count data, focusing on the top 500 most variable features [65]. Disease status was also examined as a potential group factor to evaluate its contribution to variation.

Subsequently, a differentially expressed genes (DEG) analysis was conducted comparing IBS-D patients to healthy controls using a negative binomial approach. Based on the median of ratios method, normalized counts were utilized, and genes with ≤10 counts per sample were excluded. A volcano plot was employed to visually identify DEGs, applying a log-fold change threshold of ±2 and a *p*-value cutoff of <0.05. Selected DEGs, identified by ENSEMBL ID, were then used for pathway enrichment analysis via the Reactome Analysis Tool v88 [23]. The options selected in the Reactome tool included projecting to humans and including interactors. This analysis provided a list of pathways involving at least one DEG, refined by the Reactome enrichment analysis.

### 4.4. Statistical Analysis

To discover, manage, and fit the IBS-D active module (network), we employed a combination of graph theory and structural equation modeling (SEM) to extract critical relationships (edges) and perturbations (nodes). 

Graph theory is widely used to model complex biological networks and has numerous applications in medical and biological literature related to -omics data [33,34]. SEM is a multivariate statistical approach that utilizes a system of simultaneous equations to describe the path relationships that generate the data. In this framework, a given variable (node or gene) can act as an explanatory variable in one or more equations, while serving as an outcome variable in others [31].

In our study, the network was constructed based on notable genes, excluding newly discovered transcripts from the RNA-seq analysis. 

The process was carried out in two stages as follows: (i) SEM-based gene set analysis (SEMgsa): we first used SEMgsa [25] to identify differential expression patterns; and (ii) active module fitting: next, we fitted the active IBS-D module (network) [25] based on these patterns. 

Graph theory was applied to (i) learn causal architecture using the Reactome [23] data to provide the active IBS-D module and (ii) search for network communities and paths, exploring the structure and relationships within the network.

SEM was used to (i) perform differential expression analysis (SEMgsa) for initial gene set analysis and (ii) fit the model for refining the active IBS-D network module.

It is important to note that graph theory also played a crucial role in the model-searching phase during the model-fitting step.

#### 4.4.1. SEMgsa

SEMgsa was performed to detect differential expression of individual genes between IBS-D cases and controls and to infer their biological structure by pathways interrogation of Reactome [75,76]. In this step, we followed a topology-based approach [77], which incorporated the pathway structure, improving the performance [25]. The core of the methodology is explained in Grassi et al. [25], and is based on the RICF (Residual Iterative Conditional Fitting) algorithm [78,79]. 

To run this step, normalized gene expression data and the group variable (1 = IBS-D, 0 = control) obtained by the GEO database (https://www.ncbi.nlm.nih.gov/geo/, ID = GSE146853, accessed on 2 January 2024) were provided for the analysis. In this case, the gene expression data were preliminarily transformed by a nonparanormal transformation to complain the normality assumption required by SEM [25]. In addition, a list of pathways was provided involving at least one DEG (differentially expressed gene) returned by the bioinformatic analysis performed by the Reactome database. Briefly, the output returned node-specific group effect *p*-values, and Brown’s combined *p*-values of node activation and inhibition. Node-specific *p*-values were corrected for multiple comparisons (<a = 0.25), by adjusting for the false discovery rate (FDR) used for DEGs identification. The phase of DEGs identification was also carried out in SEM-GSA to account for the topological structure of the considered pathways. The choice to set a = 0.25 was made because this work is explorative, and the aim was to provide and fit an active IBS-D module including as much a priori transcriptomic information as possible. In detail, SEMgsa returned two outputs as follows: a table reporting (i) the number of nodes for each pathway, (ii) the number of differential expression genes (DEGs) within the pathways, after multiple test corrections, (iii) pathway perturbation status (activated vs. inhibited), (iv) Brown’s combined *p*-value of pathway node activation, (v) Brown’s combined *p*-value of pathway node inhibition, (vi) the Bonferroni combined *p*-value of activation and inhibition, expressed as 2× min(activation *p*-value, inhibition *p*-value), (vii) the adjusted Bonferroni *p*-value of pathway perturbation; i.e., min(number of pathways x *p*-value in [vii]; 1), and (viii) a list with DEG names per pathway.

#### 4.4.2. Analysis of the Active IBS-D Module

We constructed and fitted an SEM-based transcriptomic active IBS-D module using differentially expressed genes (DEGs) identified through SEMgsa analysis. Initially, these DEGs, referred to as seed genes, were mapped onto the Reactome interactome—representing the union of all Reactome pathways—through a graph weighting procedure that utilized Fisher’s r-to-z transformation [80,81]. This approach tested for differences between groups in the correlation coefficients of interacting gene pairs [25]. 

To generate a perturbed reduced graph, representing the active IBS-D module, we applied a Steiner tree (ST) identification procedure based on Kou’s algorithm [82]. This process connected the seed genes to other genes (referred to as connector genes) by minimizing the total edge distance. For this purpose, the ST procedure transformed the *p*-values obtained from the r-to-z Fisher transformation by converting them into the inverse of the negative log(*p*-value), ensuring edge weights were reported within a positive continuous range. In graph theory, the ST is an acyclic graph that connects seed genes to additional nodes in the most compact manner possible [25].

After constructing the transcriptomic active IBS-D module, SEM was applied to the gene expression data to quantify and verify perturbations (status group->gene) and effects (gene-i->gene-j), denoted as β, among the genes within the module, comparing the different groups [25]. The statistical significance of these effects was assessed using two-tailed z-tests, with null hypothesis H0: β = 0, and significance was considered at an adjusted *p*-value < 0.05 [31,83,84]. SEM fitting was performed using constrained Gaussian graphical modeling (CGGM), incorporating a DAG node-wise Lasso procedure and de-biasing asymptotic inference [85].

Notably, the effects of gene-i or group perturbations on gene-j were represented as regression coefficients, which were conditionally interpretable. This means that the coefficients reflect the expected normalized change in gene-j expression per unit increase in gene-i expression (or the difference between cases and controls), holding other variables constant within the transcriptomic active IBS-D module. Specifically, negative coefficients indicated inhibition (non-activation), while positive coefficients indicated activation in regulatory terms. Similarly, negative group effects signaled downregulation (lower expression levels between cases and controls), whereas positive values indicated upregulation [83].

We fitted three models as follows: (i) Model A: the IBS-D model, evaluating edge effects specifically in IBS-D data; (ii) Model B: the control model, evaluating edge effects in controls; (iii) Model C: the node and edge perturbation model, evaluating both node and edge perturbation effects.

For Model C, a two-step procedure was conducted as follows: (1) In the first step, the group was modeled as an exogenous variable influencing all other graph nodes, and (2) in the second step, the differences in beta coefficients (edges) between the groups were estimated. This was akin to fitting separate models for cases and controls and assessing the significance of edges (direct effects) based on these differences.

Within this framework, indirect effects were also explored to identify potential gene mediators.

To enhance SEM fitting, we applied strategies such as extracting the optimal DAG by balancing model adjustment and graph sparsity and using a de-confounding process to minimize badness-of-fit measures while preserving the strongest perturbation signals present in the original data [25]. Given the exploratory nature of this study, we did not resize the DAG to exclusively reflect the biological structure of the disease based on data alone. However, our goal remained to optimize SEM fitting by considering a balance between model complexity, fitting, and perturbation.

A goodness-of-fit index, such as the standardized root mean-square residual (SRMR), was calculated to assess the model fit of the transcriptomic active IBS-D module to the data [24]. While this study is exploratory, an SRMR of less than 0.10 is generally considered an adequate fit, and values below 0.05 suggest a good fit [86].

Finally, we examined network edge changes to detect alterations in causal relationships between gene regulatory networks under different conditions. This approach is crucial for studying biological systems, as it sheds light on how gene networks shift across conditions, providing a deeper understanding of complex diseases like IBDs [87].

#### 4.4.3. Gene Communities Detection

Subsequently, a clustering procedure was carried out to identify topological gene communities using the walk-trap community detection algorithm (WTC) [25,88]. This algorithm generated the necessary number of clusters to encompass the entire input network. To assess the effectiveness of the clustering, a modularity index was calculated, which indicates the degree of separation between communities; a value close to 1 suggests well-defined community structures. Clustering was applied to both the Steiner tree graph and the optimal directed acyclic graph (DAG) obtained from the previously described fitting strategies, allowing comparison of the outcomes.

Statistical analysis was conducted using R software (version 4.3.3, R Core Team, 2023) with the SEMgraph [25], huge [80], and hgu95av2.db [89] packages (the R code is provided in the Supplemental Materials). The R/SEMgraph package offers tools for modeling complex biological systems as causal multivariate networks. The R/huge package was employed for the non-paranormal transformation of gene expression data, while the R/hgu95av2.db package was used for annotation purposes, specifically to convert Entrez identifiers into gene symbols for graphical representation of the networks.

#### 4.4.4. Communities’ Characterization

Finally, to biologically characterize the communities identified through topological clustering of the Steiner Tree and active IBS-D module, we conducted a functional enrichment analysis using the gProfiler bioinformatics tool [90]. Notably, the g: SCS algorithm [90] was employed to account for multiple testing corrections.

## 5. Conclusions

In conclusion, this study comprehensively explores the pathophysiology of IBS-D through advanced transcriptomic analysis and network-based bioinformatics approaches. Our findings confirm that IBS-D is primarily a functional disorder characterized by persistent gastrointestinal symptoms without evident structural alterations. We identified dysregulation in the complement system, which plays a crucial role in immune defense and maintaining intestinal homeostasis. This evidence underscores the importance of gut microbiota and epithelial integrity in perpetuating disease symptoms.

This study highlights critical genes, such as DUOX2 and SH2D6, which are involved in mucosal defense and immune signaling, as significant contributors to IBS-D pathophysiology. The network analysis revealed complex gene interactions and specific biological pathways, suggesting potential therapeutic targets for modulating intestinal microbiota, immune responses, and epithelial integrity.

Overall, these results advance our understanding of the molecular mechanisms underlying IBS-D and suggest promising directions for devising targeted therapeutic strategies.

## Figures and Tables

**Table 1:** Gender, age, and BMI characteristics in IBS-D and healthy patients.

	Samples	Male	Female	Age (years)	BMI (kg/m^2^)
Healthy	18	5	13	23–59	27
IBS-D	17	6	11	26–59	27

BMI: Body Mass Index.

**Table 2:** Negative binomial analysis by DESeq2 identified DEGs, comparing IBS-D versus healthy samples.

Gene_ID	ENSEMBL_ID	Gene Description	log2FC	*p*-Value
DUOX2	ENSG00000140279	dual oxidase 2	2.52	0
KLK12	ENSG00000186474	kallikrein related peptidase 12	1.38	0
SH2D6	ENSG00000152292	SH2 domain containing 6	1.26	0
MST1P2	ENSG00000186301	macrophage stimulating 1 pseudogene	−1.84	0
None	ENSG00000290851	novel transcript	−2.09	0
RPL13P12	ENSG00000215030	ribosomal protein L13 pseudogene 12	−2.8	0
HTR3C	ENSG00000178084	5-hydroxytryptamine receptor 3C	1.39	0.0001
SH2D7	ENSG00000183476	SH2 domain containing 7	1.12	0.0001
HMX3	ENSG00000188620	H6 family homeobox 3	1.11	0.0001
RPSAP15	ENSG00000237506	ribosomal protein SA pseudogene 15	1.04	0.0001
ELFN2	ENSG00000166897	extracellular leucine-rich repeat and fibronectin type III domain containing 2	1.01	0.0003
AGAP7P	ENSG00000264204	ArfGAP with GTPase domain, ankyrin repeat and PH domain 7, pseudogene	−1.91	0.0005
RPL29P4	ENSG00000230202	ribosomal protein L29 pseudogene 4	1.79	0.0006
None	ENSG00000277400	None	1.35	0.0006
DUOXA2	ENSG00000140274	dual oxidase maturation factor 2	2	0.0007
ACTG1P22	ENSG00000271615	actin gamma 1 pseudogene 22	1.16	0.0008
GTF2H2	ENSG00000145736	general transcription factor IIH subunit 2	1.03	0.0008
ATP12A	ENSG00000075673	ATPase H+/K+ transporting non-gastric alpha2 subunit	−2.01	0.0011
HTR3E	ENSG00000186038	5-hydroxytryptamine receptor 3E	1.16	0.0012
CHAT	ENSG00000070748	choline O-acetyltransferase	1.05	0.0014
MT1H	ENSG00000205358	metallothionein 1H	−1.04	0.0014
HCG4B	ENSG00000227262	HLA complex group 4B	−1.12	0.0016
PKD1P2	ENSG00000227827	polycystin 1, transient receptor potential channel interacting pseudogene 2	−2.08	0.0017
GATD3	ENSG00000160221	glutamine amidotransferase class 1 domain containing 3	1.63	0.0021
IGHV2-70	ENSG00000274576	immunoglobulin heavy variable 2–70	−1.23	0.0025
SLC13A3	ENSG00000158296	solute carrier family 13 member 3	−1.02	0.0028
IGHV5-78	ENSG00000211978	immunoglobulin heavy variable 5–78 (pseudogene)	1.4	0.003
ADGRF1	ENSG00000153292	adhesion G protein-coupled receptor F1	1.25	0.0032
IGKV2-29	ENSG00000253998	immunoglobulin kappa variable 2–29	−1.93	0.0045
FAM230I	ENSG00000178248	family with sequence similarity of 230 member, I	1.32	0.0048
None	ENSG00000287188	novel transcript, antisense to ANXA10	1.22	0.0057
ITPRID1	ENSG00000180347	ITPR interacting domain containing 1	1.02	0.0065
PI16	ENSG00000164530	peptidase inhibitor 16	−1.17	0.0067
MGAM2	ENSG00000257743	maltase-glucoamylase 2 (putative)	1.05	0.0078
None	ENSG00000230563	novel transcript	1.09	0.0111
CCNO	ENSG00000152669	cyclin O	1.04	0.0117
CHIT1	ENSG00000133063	chitinase 1	1.47	0.0136
AFF2	ENSG00000155966	ALF transcription elongation factor 2	1.28	0.0143
HERC2P3	ENSG00000290376	HERC2 pseudogene 3	1.12	0.0144
None	ENSG00000289911	novel transcript, antisense to PTP4A1	−1.06	0.0159
SFRP2	ENSG00000145423	secreted frizzled-related protein 2	−1.01	0.0178
LGSN	ENSG00000146166	lengsin, lens protein with glutamine synthetase domain	−1.67	0.0187
SULT2A1	ENSG00000105398	sulfotransferase family 2A member 1	−2.18	0.0188
None	ENSG00000274767	novel transcript, antisense CCL3L3	−1.15	0.0212
PLA2G10BP	ENSG00000254609	phospholipase A2 group XB, pseudogene	1.49	0.0214
None	ENSG00000289810	novel transcript	1.3	0.0244
None	ENSG00000224114	ribosomal protein S14 (RPS14) pseudogene	−1.31	0.0249
OLFM4	ENSG00000102837	olfactomedin 4	1.26	0.0255
CR2	ENSG00000117322	complement C3d receptor 2	1.29	0.0267
LTF	ENSG00000012223	Lactotransferrin	1.08	0.0315
None	ENSG00000213058	ribosomal protein S14 (RPS14) pseudogene	−1.64	0.0328
HSD3B2	ENSG00000203859	hydroxy-delta-5-steroid dehydrogenase, 3 beta- and steroid delta-isomerase 2	1.23	0.039
PWP2	ENSG00000241945	PWP2 small subunit processome component	1.09	0.0406
SVOPL	ENSG00000157703	SVOP like	−1	0.0481

The genes are arranged according to their *p*-value in ascending order. Abbreviations: Gene_ID: gene identification; ENSEMBL_ID: ENSEMBLE Identification; Log2FC: Log2 fold-change.

**Table 3:** Results of the SEMgsa.

Pathway	No. Nodes	No. DEGs	Perturbation Status	*p*Na	*p*Ni	*p*val
Creation of C4 and C2 activators	14	5	Down	0.999	0.000	0.001
Formation of the Early Elongation Complex	33	2	Up	0.001	0.995	0.003
Classical antibody-mediated complement activation	6	5	Down	1.000	0.002	0.004
Complement cascade	57	8	Down	0.680	0.006	0.011
Regulation of complement cascade	46	7	Down	0.694	0.007	0.014
Initial triggering of complement	22	6	Down	0.611	0.008	0.016
Negative regulation of TCF-dependent signaling by WNT ligand antagonists	15	4	Down	0.742	0.008	0.016
Digestion of dietary carbohydrate	7	2	Down	0.370	0.144	0.288
FCGR activation	12	2	Down	0.415	0.049	0.098
Role of phospholipids in phagocytosis	24	0	Down	0.306	0.231	0.462
Thyroxine biosynthesis	7	1	Up	0.230	0.600	0.460
Scavenging of heme from plasma	12	0	Down	0.587	0.131	0.261
Role of LAT2/NTAL/LAB on calcium mobilization	12	0	Up	0.298	0.372	0.597
FCERI-mediated Ca^+2^ mobilization	30	1	Up	0.177	0.495	0.353
CD22-mediated BCR regulation	5	0	Up	0.368	0.844	0.735
FCGR3A-mediated IL10 synthesis	28	2	Up	0.321	0.323	0.642
Antigen activates B-Cell Receptor (BCR), leading to the generation of second messengers	29	0	Up	0.414	0.590	0.827

The pathway mineralocorticoid biosynthesis analyzed in SEMgsa has not been included in the table because the RICF algorithm did not converge. Abbreviations: No: number; DEGs: differentially expressed genes; up: activated; down: inhibited; *p*Na: Brown’s combined *p*-value of pathway node activation; *p*Ni: Brown’s combined *p*-value of pathway node inhibition; *p*val: Bonferroni combined *p*-value of activation and inhibition, expressed as 2× min (activation *p*-value, inhibition *p*-value); ADJP: adjusted Bonferroni *p*-value of pathway perturbation; i.e., min (number of pathways × *p*-value; 1).

**Table 4:** List of differentially expressed genes (DEGs) per pathway.

Pathway	DEG 1	DEG 2	DEG 3	DEG 4	DEG 5	DEG 6	DEG 7	DEG 8
Creation of C4 and C2 activators	C1QC	C1QB	MASP1	C1S	C1R			
Formation of the Early Elongation Complex	GTF2H2	SUPT4H1						
Classical antibody-mediated complement activation	C1QA	C1QC	C1QB	C1S	C1R			
Complement cascade	C1QC	C1QB	C4BPA	MASP1	C6	C1S	C1R	C3AR1
Regulation of complement cascade	C1QC	C1QB	C4BPA	C6	C1S	C1R	C3AR1	
Initial triggering of complement	C1QC	C1QB	MASP1	C4B	C1S	C1R		
Negative regulation of TCF-dependent signaling by WNT ligand antagonists	LRP5	SFRP1	LRP5	KREMEN1				
Digestion of dietary carbohydrate	SI	MGAM						
FCGR activation	FGCR2A	CD247						
Role of phospholipids in phagocytosis								
Thyroxine biosynthesis	DUOX2							
Scavenging of heme from plasma								
Role of LAT2/NTAL/LAB on calcium mobilization								
FCERI-mediated Ca^+2^ mobilization	LCP2							
CD22-mediated BCR regulation								
FCGR3A-mediated IL10 synthesis	FGCR2A	CD247						
Antigen activates B-Cell Receptor (BCR), leading to the generation of second messengers								

**Table 5:** Results of the SEM fitting: gene effects in the IBS-D and control group.

Gene	Path	Gene	IBS-D Model					Control Model				
			β	*p*-Value	95%CI inf	95%CI Sup	A/NA	β	*p*-Value	95%CI inf	95%CI Sup	A/NA
SLC9B1	→	CA4	**0.481**	**0.016**	**0.089**	**0.874**	**A**	*−0.343*	*0.084*	*−0.732*	*0.046*	*NA*
LRP6	→	CA4	−0.251	0.21	−0.644	0.141	NA	*−0.383*	*0.054*	*−0.772*	*0.006*	*NA*
MGAM	→	SLC5A1	0.023	0.926	−0.453	0.498	A	*0.386*	*0.076*	*−0.041*	*0.812*	*A*
UBC	→	SCNN1G	**0.819**	**0**	**0.546**	**1.092**	**A**	−0.068	0.772	−0.529	0.393	NA
UBC	→	SFRP1	−0.331	0.149	−0.779	0.118	NA	0.078	0.74	−0.383	0.539	A
CA4	→	DUOX2	0.457	**0.034**	**0.034**	**0.88**	**A**	**0.808**	**0**	**0.536**	**1.08**	**A**
SLC9B1	→	AKAP1	**−0.602**	**0**	**−0.875**	**−0.33**	**NA**	0.227	0.167	−0.095	0.548	A
SLC5A1	→	AKAP1	0.093	0.468	−0.159	0.345	A	**0.619**	**0**	**0.28**	**0.959**	**A**
SCNN1G	→	AKAP1	**−0.409**	**0.004**	**−0.687**	**−0.131**	**NA**	*0.304*	*0.085*	*−0.042*	*0.649*	*A*
SFRP1	→	FZD1	**0.533**	**0.009**	**0.131**	**0.935**	A	0.356	0.106	−0.076	0.787	A

A: Activated; NA: not activated. Significant results (*p* < 0.05) are shown in bold, and suggestive results (0.05 < *p* < 0.10) in italics. β: Effect. The standardized root mean-squared residual (SRMR) for the IBS-D model was 0.212, compared to 0.224 for the control model.

**Table 6:** Results of the SEM fitting: gene differences (i.e., node perturbation by “node and edge perturbation” model) between the IBS-D and control group.

Source	Path	Gene	b	*p*-Value	95%CI inf	95%CI sup	Up/Down
Group	→	SLC9B1	−0.177	0.288	−0.503	0.149	down-expressed
Group	→	LRP6	0.101	0.547	−0.228	0.431	upregulated
Group	→	MGAM	0.266	0.102	−0.053	0.586	upregulated
*Group*	*→*	UBC	*0.271*	*0.096*	*−0.048*	*0.589*	*upregulated*
**Group**	**→**	**CA4**	**0.474**	**0.001**	**0.192**	**0.755**	**upregulated**
Group	→	SLC5A1	0.221	0.185	−0.106	0.547	upregulated
Group	→	SCNN1G	−0.247	0.11	−0.549	0.056	down-expressed
**Group**	**→**	**SFRP1**	**−0.405**	**0.009**	**−0.71**	**−0.1**	**down-expressed**
Group	→	DUOX2	0.098	0.471	−0.169	0.366	upregulated
Group	→	AKAP1	−0.039	0.788	−0.324	0.246	down-expressed
Group	→	FZD1	−0.081	0.62	−0.402	0.24	down-expressed

In bold, significant (*p* < 0.05) effects, and in italics suggestive results (0.05 < *p* < 0.10). b: effect. 95%CI: 95% confidence interval. The SRMR of the model was equal to 0.218.

**Table 7:** Results of the SEM fitting: gene effect differences between the IBS-D and control group (i.e., edge perturbation, from “node and edge perturbation” model).

Gene	Path	Gene	db	*p* Value	95%CI inf	95%CI Sup	Up/Down
**SLC9B1**	**→**	**CA4**	**0.825**	**0.003**	**0.272**	**1.377**	**upregulated**
LRP6	→	CA4	0.132	0.640	−0.421	0.684	upregulated
MGAM	→	SLC5A1	−0.363	0.265	−1.001	0.275	downregulated
**UBC**	**→**	**SCNN1G**	**0.887**	**0.001**	**0.352**	**1.423**	**upregulated**
UBC	→	SFRP1	−0.409	0.213	−1.052	0.234	downregulated
CA4	→	DUOX2	−0.351	0.171	−0.854	0.151	downregulated
**SLC9B1**	**→**	**AKAP1**	**−0.829**	**0.000**	**−1.250**	**−0.407**	**downregulated**
**SLC5A1**	**→**	**AKAP1**	**−0.526**	**0.015**	**−0.949**	**−0.103**	**downregulated**
**SCNN1G**	**→**	**AKAP1**	**−0.713**	**0.002**	**−1.156**	**−0.269**	**downregulated**
SFRP1	→	FZD1	0.178	0.555	−0.412	0.768	upregulated

In bold, the significant (*p* < 0.05) effects. Abbreviations: db: effect difference between IBS-D and control samples. 95%CI: 95% confidence interval. The SRMR of the model was equal to 0.218.

**Table 8:** Gene ontology results of ST communities. Abbreviations: BP: biological processes, Term_name: GO term name, Term_id: GO term identification, adj *p*-value: adjusted *p*-value.

Community	Source	Term Name	Term id	adj_*p*-Value
1	GO:BP	complement activation	GO:0006956	1.38 × 10^−2^
2	GO:BP	immune response-regulating cell surface receptor signaling pathway	GO:0002768	2.48 × 10^−4^
	GO:BP	regulation of immune response	GO:0050776	1.15 × 10^−3^
	GO:BP	regulation of immune system process	GO:0002682	1.15 × 10^−3^
	GO:BP	immune response-activating signaling pathway	GO:0002757	1.42 × 10^−3^
	GO:BP	immune response-regulating signaling pathway	GO:0002764	1.87 × 10^−3^
	GO:BP	activation of immune response	GO:0002253	2.72 × 10^−3^
	GO:BP	positive regulation of immune system process	GO:0002684	3.28 × 10^−3^
	GO:BP	immune system process	GO:0002376	3.82 × 10^−3^
	GO:BP	transmembrane receptor protein tyrosine kinase signaling pathway	GO:0007169	5.63 × 10^−3^
3	GO:BP	disaccharide catabolic process	GO:0046352	4.11 × 10^−5^
	GO:BP	disaccharide metabolic process	GO:0005984	4.52 × 10^−4^
	GO:BP	oligosaccharide catabolic process	GO:0009313	7.19 × 10^−4^
	GO:BP	oligosaccharide metabolic process	GO:0009311	1.51 × 10^−2^
4	-	-	-	-
5	-	-	-	-
6	GO:BP	midbrain dopaminergic neuron differentiation	GO:1904948	7.82 × 10^−7^
	GO:BP	dopaminergic neuron differentiation	GO:0071542	1.83 × 10^−5^
	GO:BP	canonical Wnt signaling pathway	GO:0060070	2.89 × 10^−5^
	GO:BP	Wnt signaling pathway involved in somitogenesis	GO:0090244	1.09 × 10^−4^
	GO:BP	Wnt signaling pathway	GO:0016055	1.40 × 10^−4^
	GO:BP	cell-cell signaling by wnt	GO:0198738	1.43 × 10^−4^
	GO:BP	midbrain development	GO:0030901	2.15 × 10^−4^
	GO:BP	negative regulation of canonical Wnt signaling pathway	GO:0090090	7.02 × 10^−4^
	GO:BP	planar cell polarity pathway involved in neural tube closure	GO:0090179	9.94 × 10^−4^
	GO:BP	regulation of establishment of planar polarity involved in neural tube closure	GO:0090178	1.19 × 10^−3^
7	GO:BP	visual system development	GO:0150063	3.51 × 10^−4^
	GO:BP	sensory system development	GO:0048880	3.78 × 10^−4^
	GO:BP	tube morphogenesis	GO:0035239	4.28 × 10^−4^
	GO:BP	Norrin signaling pathway	GO:0110135	1.11 × 10^−3^
	GO:BP	tube development	GO:0035295	1.60 × 10^−3^
	GO:BP	extracellular matrix-cell signaling	GO:0035426	1.85 × 10^−3^
	GO:BP	Wnt signaling pathway, calcium modulating pathway	GO:0007223	1.85 × 10^−3^
	GO:BP	retinal blood vessel morphogenesis	GO:0061304	2.77 × 10^−3^
	GO:BP	blood vessel morphogenesis	GO:0048514	2.96 × 10^−3^
	GO:BP	cellular response to retinoic acid	GO:0071300	3.32 × 10^−3^
8	GO:BP	complement activation	GO:0006956	1.47 × 10^−19^
	GO:BP	humoral immune response	GO:0006959	5.60 × 10^−14^
	GO:BP	complement activation, classical pathway	GO:0006958	9.30 × 10^−12^
	GO:BP	humoral immune response mediated by circulating immunoglobulin	GO:0002455	5.90 × 10^−11^
	GO:BP	immune effector process	GO:0002252	5.40 × 10^−10^
	GO:BP	positive regulation of immune response	GO:0050778	6.93 × 10^−10^
	GO:BP	regulation of immune response	GO:0050776	4.37 × 10^−9^
	GO:BP	innate immune response	GO:0045087	9.93 × 10^−9^
	GO:BP	positive regulation of immune system process	GO:0002684	2.19 × 10^−8^
	GO:BP	synapse pruning	GO:00998883	3.30 × 10^−5^

**Table 9:** Gene ontology results of IBS-D module. Abbreviations: ‘community number’, BP: biological processes, Term_name: GO term name, Term_id: GO term identification, adj_*p*-value: adjusted -value.

Communities	Source	Term_Name	Term_id	adj_*p*_Value
1	-	-	-	-
2	GO:BP	sodium ion transmembrane transport	GO:0035725	3.20 × 10^−2^
	GO:BP	maltose catabolic processes	GO:0090178	4.95 × 10^−2^
	GO:BP	starch metabolic processes	GO:0090177	4.95 × 10^−2^
	GO:BP	starch catabolic processes	GO:0090175	4.95 × 10^−2^
	GO:BP	dextrin catabolic processes	GO:1904948	4.95 × 10^−2^
3	GO:BP	planar cell polarity pathway involved in neural tube closure	GO:0090179	9.94 × 10^−4^
	GO:BP	regulation of establishment of planar polarity involved in neural tube closure	GO:0090178	1.19 × 10^−3^
	GO:BP	establishment of planar polarity involved in neural tube closure	GO:0090177	1.41 × 10^−3^
	GO:BP	regulation of establishment of planar polarity	GO:0090175	1.90 × 10^−3^
	GO:BP	midbrain dopaminergic neuron differentiation	GO:1904948	1.90 × 10^−3^
	GO:BP	establishment of planar polarity of embryonic epithelium	GO:0042249	1.90 × 10^−3^
	GO:BP	establishment of tissue polarity	GO:0007164	1.27 × 10^−2^
	GO:BP	establishment of planar polarity	GO:0001736	1.27 × 10^−2^
	GO:BP	dopaminergic neuron differentiation	GO:0071542	1.48 × 10^−2^
	GO:BP	Wnt signaling pathway, planar cell polarity pathway	GO:0060071	2.21 × 10^−2^

## Data Availability

The datasets used and/or analyzed during the current study are available from the corresponding author upon reasonable request.

## Supplementary Materials

The following supporting information can be downloaded at: https://www.mdpi.com/article/10.3390/ijms25179322/s1.
