# Supplementary material for: Transcriptomic Module Discovery of Diarrhea-Predominant Irritable Bowel Syndrome: A Causal Network Inference Approach
Source: Int J Mol Sci. 2024 Aug 28;25(17):9322. doi: 10.3390/ijms25179322 (PMC11394741; doi:10.3390/ijms25179322)
Supplement: Supplementary file 1 [file ijms-25-09322-s001.zip › Table S1.pdf]

Table S1. RNA-seq preprocessing results for the 164 GEO FastQ files. Abbreviations: M_Seqs: Millions of Sequences; %_GC: GC percentage; rRNA%: Ribosomal RNA percentage; R1 and R2: Read 1 and 2 of the paired-end.

Number	DATASET_ID	Organ	Sample Name	Healthy	Gender	M Seqs	% GC	rRNA%
1	GSE166869	Jejunum	SRR13723815_R1	Healthy	female	17.8	58%	75.8%
2	GSE166869	Jejunum	SRR13723815_R2	Healthy	female	17.8	57%	75.8%
3	GSE166869	Jejunum	SRR13723816_R1	Healthy	male	41.7	52%	57.1%
4	GSE166869	Jejunum	SRR13723816_R2	Healthy	male	41.7	53%	57.1%
5	GSE166869	Jejunum	SRR13723817_R1	Healthy	male	32.6	50%	47.5%
6	GSE166869	Jejunum	SRR13723817_R2	Healthy	male	32.6	52%	47.5%
7	GSE166869	Jejunum	SRR13723822_R1	Healthy	male	33.2	50%	93.4%
8	GSE166869	Jejunum	SRR13723822_R2	Healthy	male	33.2	51%	93.4%
9	GSE166869	Jejunum	SRR13723832_R1	Healthy	female	26.6	55%	72.9%
10	GSE166869	Jejunum	SRR13723832_R2	Healthy	female	26.6	56%	72.9%
11	GSE166869	Jejunum	SRR13723846_R1	Healthy	male	17.6	50%	22.7%
12	GSE166869	Jejunum	SRR13723846_R2	Healthy	male	17.6	50%	22.7%
13	GSE166869	Jejunum	SRR13723847_R1	Healthy	male	36.6	49%	86.6%
14	GSE166869	Jejunum	SRR13723847_R2	Healthy	male	36.6	51%	86.6%
15	GSE166869	Jejunum	SRR13723848_R1	Healthy	male	17.8	57%	74.2%
16	GSE166869	Jejunum	SRR13723848_R2	Healthy	male	17.8	57%	74.2%
17	GSE166869	Jejunum	SRR13723849_R1	Healthy	male	22.6	51%	90.3%
18	GSE166869	Jejunum	SRR13723849_R2	Healthy	male	22.6	53%	90.3%
19	GSE166869	Jejunum	SRR13723851_R1	Healthy	female	37.9	50%	88.1%
20	GSE166869	Jejunum	SRR13723851_R2	Healthy	female	37.9	50%	88.1%
21	GSE166869	Jejunum	SRR13723853_R1	Healthy	female	3.1	58%	58.1%
22	GSE166869	Jejunum	SRR13723853_R2	Healthy	female	3.1	57%	58.1%
23	GSE166869	Jejunum	SRR13723854_R1	Healthy	female	13.8	50%	73.9%
24	GSE166869	Jejunum	SRR13723854_R2	Healthy	female	13.8	51%	73.9%
25	GSE166869	Jejunum	SRR13723880_R1	Healthy	male	29.6	47%	85.5%
26	GSE166869	Jejunum	SRR13723880_R2	Healthy	male	29.6	47%	85.5%
27	GSE166869	Jejunum	SRR13723818_R1	IBS-D	female	27.3	56%	93.4%
28	GSE166869	Jejunum	SRR13723818_R2	IBS-D	female	27.3	56%	93.4%
29	GSE166869	Jejunum	SRR13723819_R1	IBS-D	male	37.4	54%	62.8%
30	GSE166869	Jejunum	SRR13723819_R2	IBS-D	male	37.4	54%	62.8%
31	GSE166869	Jejunum	SRR13723825_R1	IBS-D	male	19.3	55%	93.3%
32	GSE166869	Jejunum	SRR13723825_R2	IBS-D	male	19.3	56%	93.3%
33	GSE166869	Jejunum	SRR13723828_R1	IBS-D	male	12.2	59%	85.2%
34	GSE166869	Jejunum	SRR13723828_R2	IBS-D	male	12.2	59%	85.2%
35	GSE166869	Jejunum	SRR13723829_R1	IBS-D	female	24.9	58%	92.4%
36	GSE166869	Jejunum	SRR13723829_R2	IBS-D	female	24.9	58%	92.4%
37	GSE166869	Jejunum	SRR13723850_R1	IBS-D	male	7.4	60%	70.3%
38	GSE166869	Jejunum	SRR13723850_R2	IBS-D	male	7.4	60%	70.3%
39	GSE166869	Jejunum	SRR13723861_R1	IBS-D	male	37.9	49%	93.4%
40	GSE166869	Jejunum	SRR13723861_R2	IBS-D	male	37.9	49%	93.4%
41	GSE166869	Jejunum	SRR13723863_R1	IBS-D	male	24.8	53%	75.4%
42	GSE166869	Jejunum	SRR13723863_R2	IBS-D	male	24.8	54%	75.4%
43	GSE166869	Jejunum	SRR13723865_R1	IBS-D	female	26.2	52%	91.2%
44	GSE166869	Jejunum	SRR13723865_R2	IBS-D	female	26.2	53%	91.2%
45	GSE166869	Jejunum	SRR13723867_R1	IBS-D	male	20.9	56%	79.9%
46	GSE166869	Jejunum	SRR13723867_R2	IBS-D	male	20.9	56%	79.9%
47	GSE166869	Duodenum	SRR13723824_R1	IBS-D	male	23.6	55%	99.2%

48	GSE166869	Duodenum	SRR13723824_R2	IBS-D	male	23.6	56%	99.2%
49	GSE166869	Duodenum	SRR13723827_R1	IBS-D	male	6.8	61%	86.8%
50	GSE166869	Duodenum	SRR13723827_R2	IBS-D	male	6.8	59%	86.8%
51	GSE166869	Duodenum	SRR13723833_R1	IBS-D	male	24.2	55%	96.3%
52	GSE166869	Duodenum	SRR13723833_R2	IBS-D	male	24.2	56%	96.3%
53	GSE166869	Duodenum	SRR13723836_R1	IBS-D	female	28.9	56%	85.1%
54	GSE166869	Duodenum	SRR13723836_R2	IBS-D	female	28.9	56%	85.1%
55	GSE166869	Duodenum	SRR13723837_R1	IBS-D	female	23.7	55%	97.0%
56	GSE166869	Duodenum	SRR13723837_R2	IBS-D	female	23.7	56%	97.0%
57	GSE166869	Duodenum	SRR13723859_R1	IBS-D	male	25.8	52%	63.6%
58	GSE166869	Duodenum	SRR13723859_R2	IBS-D	male	25.8	53%	63.6%
59	GSE166869	Duodenum	SRR13723860_R1	IBS-D	male	24.8	53%	88.7%
60	GSE166869	Duodenum	SRR13723860_R2	IBS-D	male	24.8	53%	88.7%
61	GSE166869	Duodenum	SRR13723862_R1	IBS-D	male	17.8	53%	88.8%
62	GSE166869	Duodenum	SRR13723862_R2	IBS-D	male	17.8	54%	88.8%
63	GSE166869	Duodenum	SRR13723864_R1	IBS-D	female	33	56%	97.0%
64	GSE166869	Duodenum	SRR13723864_R2	IBS-D	female	33	56%	97.0%
65	GSE166869	Duodenum	SRR13723866_R1	IBS-D	male	32.2	51%	93.2%
66	GSE166869	Duodenum	SRR13723866_R2	IBS-D	male	32.2	52%	93.2%
67	GSE166869	Duodenum	SRR13723874_R1	IBS-D	female	14.6	57%	91.1%
68	GSE166869	Duodenum	SRR13723874_R2	IBS-D	female	14.6	56%	91.1%
69	GSE166869	Duodenum	SRR13723831_R1	Healthy	female	11.9	59%	94.1%
70	GSE166869	Duodenum	SRR13723831_R2	Healthy	female	11.9	59%	94.1%
71	GSE166869	Duodenum	SRR13723840_R1	Healthy	female	28.2	53%	70.6%
72	GSE166869	Duodenum	SRR13723840_R2	Healthy	female	28.2	54%	70.6%
73	GSE166869	Duodenum	SRR13723852_R1	Healthy	female	6.3	59%	74.6%
74	GSE166869	Duodenum	SRR13723852_R2	Healthy	female	6.3	58%	74.6%
75	GSE166869	Duodenum	SRR13723868_R1	Healthy	male	17.9	51%	58.7%
76	GSE166869	Duodenum	SRR13723868_R2	Healthy	male	17.9	52%	58.7%
77	GSE166869	Duodenum	SRR13723869_R1	Healthy	female	36.4	50%	46.7%
78	GSE166869	Duodenum	SRR13723869_R2	Healthy	female	36.4	51%	46.7%
79	GSE166869	Duodenum	SRR13723870_R1	Healthy	male	31.2	49%	86.9%
80	GSE166869	Duodenum	SRR13723870_R2	Healthy	male	31.2	51%	86.9%
81	GSE166869	Duodenum	SRR13723871_R1	Healthy	male	26.1	51%	48.7%
82	GSE166869	Duodenum	SRR13723871_R2	Healthy	male	26.1	52%	48.7%
83	GSE166869	Duodenum	SRR13723872_R1	Healthy	female	24.6	51%	42.7%
84	GSE166869	Duodenum	SRR13723872_R2	Healthy	female	24.6	51%	42.7%
85	GSE166869	Duodenum	SRR13723873_R1	Healthy	male	37.9	50%	83.6%
86	GSE166869	Duodenum	SRR13723873_R2	Healthy	male	37.9	51%	83.6%
87	GSE166869	Duodenum	SRR13723875_R1	Healthy	male	32.3	52%	92.0%
88	GSE166869	Duodenum	SRR13723875_R2	Healthy	male	32.3	53%	92.0%
89	GSE166869	Duodenum	SRR13723876_R1	Healthy	male	32.4	54%	92.0%
90	GSE166869	Duodenum	SRR13723876_R2	Healthy	male	32.4	54%	92.0%
91	GSE166869	Duodenum	SRR13723882_R1	Healthy	male	44.7	50%	47.0%
92	GSE166869	Duodenum	SRR13723882_R2	Healthy	male	44.7	51%	47.0%
93	GSE166869	Duodenum	SRR13723883_R1	Healthy	male	36.7	51%	54.0%
94	GSE166869	Duodenum	SRR13723883_R2	Healthy	male	36.7	52%	54.0%
95	GSE146853	Colon	SRR13081831_R1	IBS-D	male	36	0.49	16.7%
96	GSE146853	Colon	SRR13081831_R2	IBS-D	male	36	0.49	16.7%
97	GSE146853	Colon	SRR13081835_R1	IBS-D	male	37.8	50%	13.5%
98	GSE146853	Colon	SRR13081835_R2	IBS-D	male	37.8	50%	13.5%
99	GSE146853	Colon	SRR13081837_R1	IBS-D	male	41.4	50%	11.2%

100	GSE146853	Colon	SRR13081837_R2	IBS-D	male	41.4	50%	11.2%
101	GSE146853	Colon	SRR13081838_R1	IBS-D	male	40.8	50%	12.1%
102	GSE146853	Colon	SRR13081838_R2	IBS-D	male	40.8	50%	12.1%
103	GSE146853	Colon	SRR13081843_R1	IBS-D	male	48.6	49%	11.5%
104	GSE146853	Colon	SRR13081843_R2	IBS-D	male	48.6	49%	11.5%
105	GSE146853	Colon	SRR13081847_R1	IBS-D	male	47.5	49%	15.6%
106	GSE146853	Colon	SRR13081847_R2	IBS-D	male	47.5	49%	15.6%
107	GSE146853	Colon	SRR11294063_R1	IBS-D	female	43	50%	13.3%
108	GSE146853	Colon	SRR11294063_R2	IBS-D	female	43	50%	13.3%
109	GSE146853	Colon	SRR11294066_R1	IBS-D	female	42.2	49%	18.7%
110	GSE146853	Colon	SRR11294066_R2	IBS-D	female	42.2	49%	18.7%
111	GSE146853	Colon	SRR11294067_R1	IBS-D	female	29.9	50%	12.5%
112	GSE146853	Colon	SRR11294067_R2	IBS-D	female	29.9	50%	12.5%
113	GSE146853	Colon	SRR11294068_R1	IBS-D	female	40.3	50%	12.9%
114	GSE146853	Colon	SRR11294068_R2	IBS-D	female	40.3	50%	12.9%
115	GSE146853	Colon	SRR11294070_R1	IBS-D	female	41.5	49%	18.2%
116	GSE146853	Colon	SRR11294070_R2	IBS-D	female	41.5	49%	18.2%
117	GSE146853	Colon	SRR11294072_R1	IBS-D	female	36.8	50%	11.3%
118	GSE146853	Colon	SRR11294072_R2	IBS-D	female	36.8	50%	11.3%
119	GSE146853	Colon	SRR12926783_R1	IBS-D	female	67.3	49%	16.9%
120	GSE146853	Colon	SRR12926783_R2	IBS-D	female	67.3	49%	16.9%
121	GSE146853	Colon	SRR12926784_R1	IBS-D	female	29.9	49%	18.4%
122	GSE146853	Colon	SRR12926784_R2	IBS-D	female	29.9	49%	18.4%
123	GSE146853	Colon	SRR12926785_R1	IBS-D	female	33.8	49%	21.2%
124	GSE146853	Colon	SRR12926785_R2	IBS-D	female	33.8	49%	21.2%
125	GSE146853	Colon	SRR12926786_R1	IBS-D	female	50.1	50%	14.2%
126	GSE146853	Colon	SRR12926786_R2	IBS-D	female	50.1	50%	14.2%
127	GSE146853	Colon	SRR12926787_R1	IBS-D	female	50.6	49%	11.3%
128	GSE146853	Colon	SRR12926787_R2	IBS-D	female	50.6	49%	11.3%
129	GSE146853	Colon	SRR13081833_R1	Healthy	male	43.7	50%	13.4%
130	GSE146853	Colon	SRR13081833_R2	Healthy	male	43.7	50%	13.4%
131	GSE146853	Colon	SRR13081840_R1	Healthy	male	33.4	50%	14.6%
132	GSE146853	Colon	SRR13081840_R2	Healthy	male	33.4	50%	14.6%
133	GSE146853	Colon	SRR13081845_R1	Healthy	male	53.1	50%	14.3%
134	GSE146853	Colon	SRR13081845_R2	Healthy	male	53.1	50%	14.3%
135	GSE146853	Colon	SRR13081849_R1	Healthy	male	44.1	50%	14.7%
136	GSE146853	Colon	SRR13081849_R2	Healthy	male	44.1	49%	14.7%
137	GSE146853	Colon	SRR13081851_R1	Healthy	male	37	50%	21.7%
138	GSE146853	Colon	SRR13081851_R2	Healthy	male	37	50%	21.7%
139	GSE146853	Colon	SRR11294080_R1	Healthy	female	28.3	49%	17.2%
140	GSE146853	Colon	SRR11294080_R2	Healthy	female	28.3	48%	17.2%
141	GSE146853	Colon	SRR11294081_R1	Healthy	female	41.1	49%	13.9%
142	GSE146853	Colon	SRR11294081_R2	Healthy	female	41.1	49%	13.9%
143	GSE146853	Colon	SRR11294082_R1	Healthy	female	38.9	49%	13.1%
144	GSE146853	Colon	SRR11294082_R2	Healthy	female	38.9	49%	13.1%
145	GSE146853	Colon	SRR11294083_R1	Healthy	female	67.3	49%	16.9%
146	GSE146853	Colon	SRR11294083_R2	Healthy	female	67.3	49%	16.9%

147	GSE146853	Colon	SRR11294084_R1	Healthy	female	29.9	49%	18.4%
148	GSE146853	Colon	SRR11294084_R2	Healthy	female	29.9	49%	18.4%
149	GSE146853	Colon	SRR11294085_R1	Healthy	female	33.8	49%	21.2%
150	GSE146853	Colon	SRR11294085_R2	Healthy	female	33.8	49%	21.2%
151	GSE146853	Colon	SRR11294086_R1	Healthy	female	50.1	50%	14.2%
152	GSE146853	Colon	SRR11294086_R2	Healthy	female	50.1	50%	14.2%
153	GSE146853	Colon	SRR11294087_R1	Healthy	female	50.6	49%	11.3%
154	GSE146853	Colon	SRR11294087_R2	Healthy	female	50.6	49%	11.3%
155	GSE146853	Colon	SRR12926794_R1	Healthy	female	37.8	48%	13.6%
156	GSE146853	Colon	SRR12926794_R2	Healthy	female	37.8	48%	13.6%
157	GSE146853	Colon	SRR12926795_R1	Healthy	female	32.5	50%	19.7%
158	GSE146853	Colon	SRR12926795_R2	Healthy	female	32.5	50%	19.7%
159	GSE146853	Colon	SRR12926796_R1	Healthy	female	68.1	50%	13.3%
160	GSE146853	Colon	SRR12926796_R2	Healthy	female	68.1	50%	13.3%
161	GSE146853	Colon	SRR12926797_R1	Healthy	female	34.8	50%	15.9%
162	GSE146853	Colon	SRR12926797_R2	Healthy	female	34.8	50%	15.9%
163	GSE146853	Colon	SRR12926798_R1	Healthy	female	28.3	48%	14.1%
164	GSE146853	Colon	SRR12926798_R2	Healthy	female	28.3	48%	14.1%
