# Supplementary material for: Transcriptomic Module Discovery of Diarrhea-Predominant Irritable Bowel Syndrome: A Causal Network Inference Approach
Source: Int J Mol Sci. 2024 Aug 28;25(17):9322. doi: 10.3390/ijms25179322 (PMC11394741; doi:10.3390/ijms25179322)
Supplement: Supplementary file 1 [file ijms-25-09322-s001.zip › Table S2.pdf]

Table S2. Reactome pathways identified by 54 DEGs. Reactome pathways having at least 1 Entities found had been selected.

Pathway name	Curated found	Curated Total	Species name
Classical antibody-mediated complement activation	2	97	Homo sapiens
Complement cascade	3	156	Homo sapiens
Creation of C4 and C2 activators	2	111	Homo sapiens
Digestion of dietary carbohydrate	1	27	Homo sapiens
FCERI mediated Ca+2 mobilization	2	129	Homo sapiens
FCGR activation	2	103	Homo sapiens
FCGR3A-mediated IL10 synthesis	2	141	Homo sapiens
Formation of the Early Elongation Complex	1	34	Homo sapiens
Formation of the HIV-1 Early Elongation Complex	1	34	Homo sapiens
Initial triggering of complement	2	120	Homo sapiens
Metal sequestration by antimicrobial proteins	1	13	Homo sapiens
Mineralocorticoid biosynthesis	1	20	Homo sapiens
Mtb iron assimilation by chelation	1	17	Homo sapiens
Negative regulation of TCF-dependent signaling by WNT ligand antagonists	1	15	Homo sapiens
Regulation of Complement cascade	3	139	Homo sapiens
Role of LAT2/NTAL/LAB on calcium mobilization	2	107	Homo sapiens
Role of phospholipids in phagocytosis	2	129	Homo sapiens
Scavenging of heme from plasma	2	106	Homo sapiens
Thyroxine biosynthesis	1	29	Homo sapiens
