# Supplementary material for: Transcriptomic Module Discovery of Diarrhea-Predominant Irritable Bowel Syndrome: A Causal Network Inference Approach
Source: Int J Mol Sci. 2024 Aug 28;25(17):9322. doi: 10.3390/ijms25179322 (PMC11394741; doi:10.3390/ijms25179322)
Supplement: Supplementary file 1 [file ijms-25-09322-s001.zip › Table S3.pdf]

Table S3. Genes appertaining to Strainer Tree module communities.

Community N°	Genes ID									
1	C6	CD59	SPTBN2							
2	SOS1	CD247	PIK3R1	FCGR2A	PIK3CB	LCP2	PAK2	UBC	SCNN1G	AKAP1
3	MGAM	SLC5A1	SI							
4	SLC9B1	CA4	DUOX2							
5	RAD23B	GTF2H2	TBP	SUPT4H1						
6	KREMEN1	LRP6	SFRP1	FZD1						
7	WNT6	CD3D	FNBP1	FZD4	LRP5	WNT5A	GNB3	C3AR1	C3	
8	MASP1	COLEC11	C1S	C1QA	C4B	C1R	C1QB	C1QC	C4BPB	C4BPA
