# Supplementary figures and images for: Transcriptomic Module Discovery of Diarrhea-Predominant Irritable Bowel Syndrome: A Causal Network Inference Approach
Source: Int J Mol Sci. 2024 Aug 28;25(17):9322. doi: 10.3390/ijms25179322 (PMC11394741; doi:10.3390/ijms25179322)

**Figure S1.** 2-D plot of PCA of RNA-seq Samples. Disease status stratification: IBS-D and Healthy.

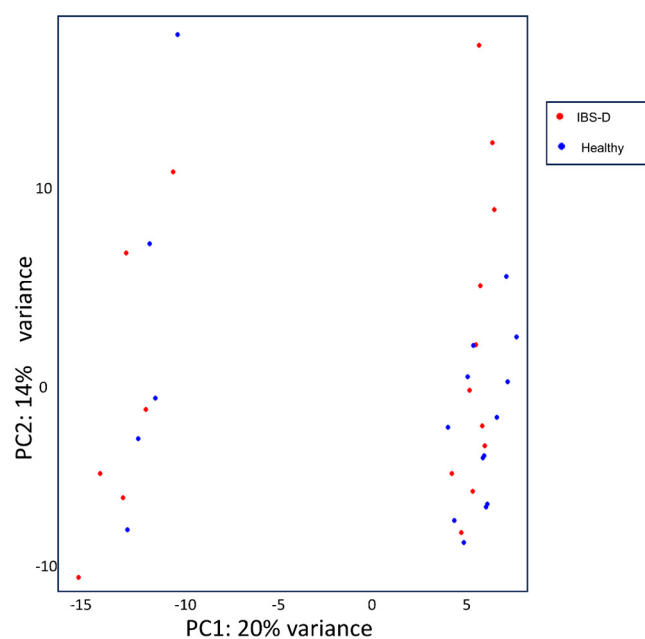

Supplement: Supplementary file 1 [file ijms-25-09322-s001.zip › Figure S1.pdf]
